# Performance similarities predict collective benefits in dyadic and triadic joint visual search

**DOI:** 10.1371/journal.pone.0191179

**Published:** 2018-01-12

**Authors:** Basil Wahn, Artur Czeszumski, Peter König

**Affiliations:** 1 Institute of Cognitive Science, University of Osnabrück, Osnabrück, Germany; 2 Department of Neurophysiology and Pathophysiology, Center of Experimental Medicine, University Medical Center Hamburg-Eppendorf, Hamburg, Germany; Tsinghua University, CHINA

## Abstract

When humans perform tasks together, they may reach a higher performance in comparison to the best member of a group (i.e., a collective benefit). Earlier research showed that interindividual performance similarities predict collective benefits for several joint tasks. Yet, researchers did not test whether this is the case for joint visuospatial tasks. Also, researchers did not investigate whether dyads and triads reach a collective benefit when they are forbidden to exchange any information while performing a visuospatial task. In this study, participants performed a joint visual search task either alone, in dyads, or in triads, and were not allowed to exchange any information while doing the task. We found that dyads reached a collective benefit. Triads did outperform their best individual member and dyads—yet, they did not outperform the best dyad pairing within the triad. In addition, similarities in performance significantly predicted the collective benefit for dyads *and* triads. Furthermore, we find that the dyads’ and triads’ search performances closely match a simulated performance based on the individual search performances, which assumed that members of a group act independently. Overall, the present study supports the view that performance similarities predict collective benefits in joint tasks. Moreover, it provides a basis for future studies to investigate the benefits of exchanging information between co-actors in joint visual search tasks.

## Introduction

In daily life, humans often perform tasks together to achieve a shared goal [1–3]. Examples are humans carrying a table together [1], searching for a friend in a crowd [4], or playing team sports such as soccer or basketball. Depending on how groups perform these tasks, they may reach a higher performance compared to the best group member’s individual performance (i.e., a “collective benefit” [5, 6]).

Collective benefits have been investigated in a wide variety of tasks such as joint visuomotor tasks [7–14], joint visuospatial tasks [4, 15–19], joint memory [20, 21], or joint perceptual decision-making tasks [5, 22–27]. Researchers found several factors that affect whether groups can outperform individuals and to what extent. In particular, they found that the more similarly well group members perform a task alone, the higher the collective benefit. This finding was reported for joint perceptual decision-making tasks [5, 28] and a joint visuomotor task [14]. In these earlier studies, the calculation of the similarities of the individual performances is based on typical performance measures in the investigated tasks. For instance, in a study investigating a joint visuomotor task [14], the individual averaged trial completion time by each member of a group was used for calculating a similarity score. In particular, the slower trial completion time was divided by the faster trial completion time, yielding a value between zero and one with a value close to one indicating a high similarity of the individual performances. To date, however, researchers did not test whether similarities between the individual performances predict collective benefits in joint visuospatial tasks. Given earlier findings on joint perceptual decision-making [5] and joint visuomotor tasks [14], such a finding could provide converging evidence that similarities in the individual performances are a general predictor for collective benefits in joint tasks. Hence, one goal of the present study is to test whether similarities in the individual performances predict collective benefits also for joint visuospatial tasks.

With regard to joint visuospatial tasks, researchers often investigated joint performance in a joint visual search task [4, 15, 16, 18]. In a joint visual search task, dyads jointly search for a target among distractors on a computer screen. Typically, in half of the trials a target is present and participants’ task is to indicate whether a target is present or absent. In a previous study [4], researchers compared joint visual search task performance, i.e., how accurate and fast dyads searched, between conditions that varied the information that co-actors received. That is, co-actors were allowed to verbally communicate and/or saw a cursor on the screen, indicating where their search partner was looking, or received no information about their co-actor. In addition, a separate set of participants performed the search task alone. They found that dyads searched faster than individuals in all conditions. Importantly, when dyads received no information about their co-actor, they also outperformed individuals. That is, co-actors without any means to exchange information (neither verbal information nor gaze information) still outperformed individuals. Due to the between-subject design, however, researchers [4] could not test whether dyads attained a collective benefit as a comparison between the best member’s performance and the joint performance was not possible. Yet, given the large effect sizes in the previous study, it is likely that dyads would have reached a collective benefit. Another goal of the present study is to replicate this finding by Brennan and colleagues [4] and extent it by testing whether dyads also outperform the best member in the group (i.e., reach a collective benefit). Moreover, to date, researchers did not investigate how triads perform in comparison to individuals and dyads. In the present study, we therefore also test whether triads outperform their best individual member, outperform dyads, and importantly, whether triads also outperform their best dyad pairing in the triad. Note, for the latter comparison, we extent the definition of a collective benefit as used in earlier studies [5, 19] to larger group sizes. That is, in line with the definition of collective benefits for dyads [5], we compare the triad’s performance with the best dyad pairing within the triad to test whether triads also attain a collective benefit.

As in the previous study by Brennan and colleagues [4], dyads and triads do not exchange any information while performing the joint visual search task in the present study. That is, they have no means to communicate any search strategy with their co-actor to ease the joint search (e.g., a division of labour strategy [4]). Given there is no exchange of information possible between co-actors, we predict that dyads and triads do act independently while performing the joint search (i.e., do not collaborate). To test whether the co-actors of dyads and triads actually perform the search independently, we aim to compare the actual joint performance to a simulated joint performance, which assumes that co-actors in a group act independently [15, 19]. Such a simulated joint performance has been used in earlier studies to test whether members of a dyad do collaborate [15, 19]. That is, if dyads surpassed the simulated joint performance, researchers inferred that members of a dyad did collaborate (e.g., used a division of labour strategy). Importantly, in one these earlier studies [19], findings showed that dyads can already reach a collective benefit in a joint visuospatial task even without a division of labour strategy. That is, due to the mere fact that two people perform a task and are not collaborating, dyads can already attain a collective benefit. Given these findings [19] and if dyads and triads do act independently as expected, we still predict to find collective benefits in the present study.

**Fig 1:**
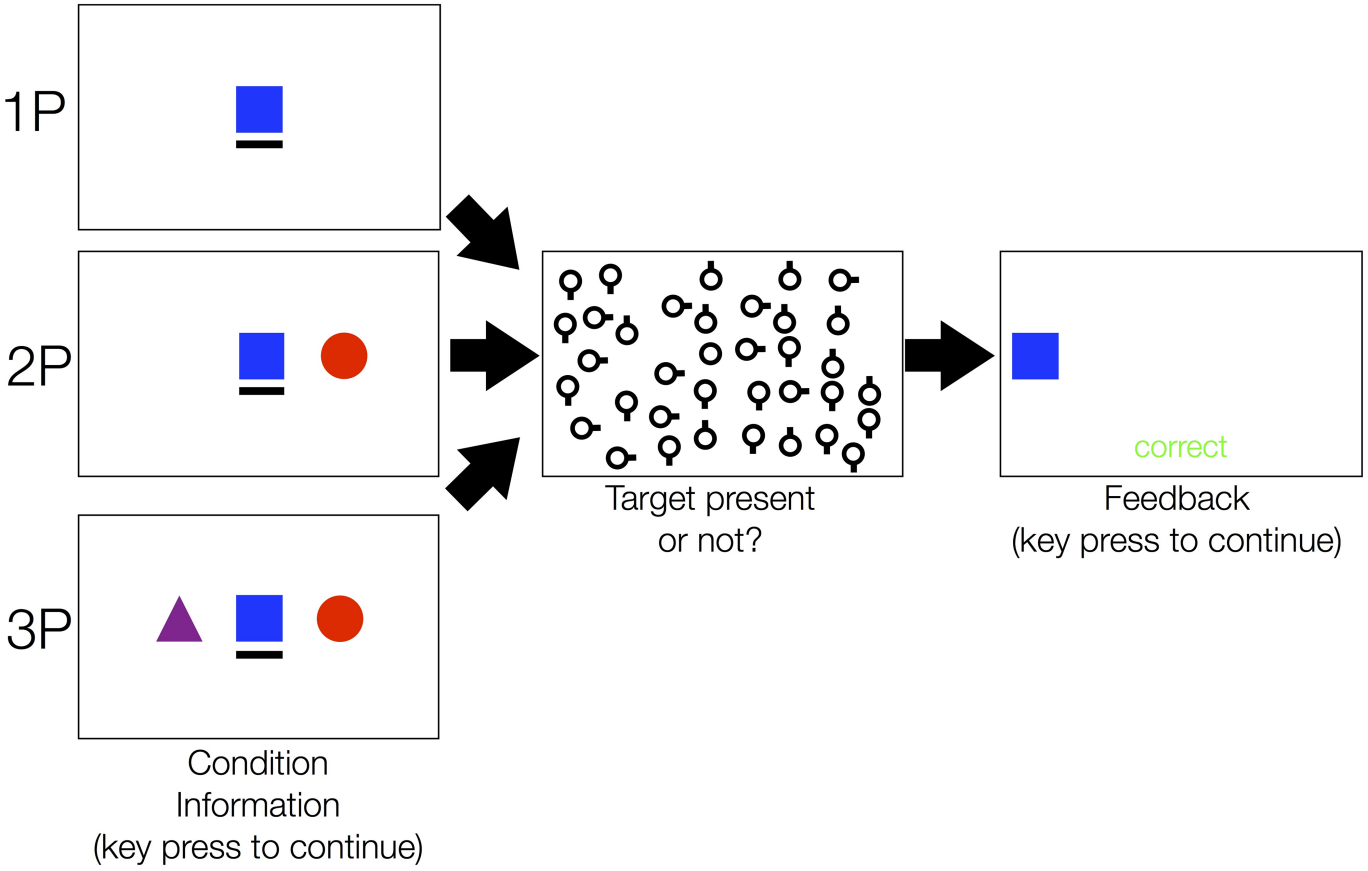
Trial overview. Prior to the experiment, each participant was assigned to one avatar (either a blue square, a red circle, or a violet triangle). Here, a trial overview from the perspective of a participant with the blue square avatar is shown. Before a trial started, participants saw whether they would perform the task alone (top row), in dyads (middle row), or in triads (bottom row). The black underscore indicated the identity of the participant (i.e., in this case the blue square). Hence, members of a triad were always aware whether they searched alone, in a dyad, or in a triad. In the search task, 36 objects are always displayed. In half of the trials, one of these objects was the target object (i.e., a circle) among distractor objects (i.e., circles with an antenna attached to it). Participants were required to indicate whether a target was present or absent. They were instructed to perform the search quickly but still accurately. In all conditions, only the first response counted to performance and the search was terminated once a response was given. Then, participants received performance feedback and were informed which co-actor responded.

In sum, we aim to replicate the finding by Brennan and colleagues [4] that dyads do outperform individuals in a joint visual search task and extend this finding by testing whether they also do reach a collective benefit. Furthermore, we test whether triads also reach a collective benefit, outperform dyads, and whether they outperform the best dyad pairing within the triad. In addition, given that co-actors cannot exchange any information during a trial, we test whether the joint search performance matches a simulated joint performance, which assumes that co-actors act independently. Finally, we test whether similarities in the individual performance are a predictor of the collective benefit both for dyads or triads. To investigate these goals, participants performed a joint visual search task, in which they searched for a target among distractors either alone, in dyads, or in triads (Fig 1).

## Results

**Fig 2:**
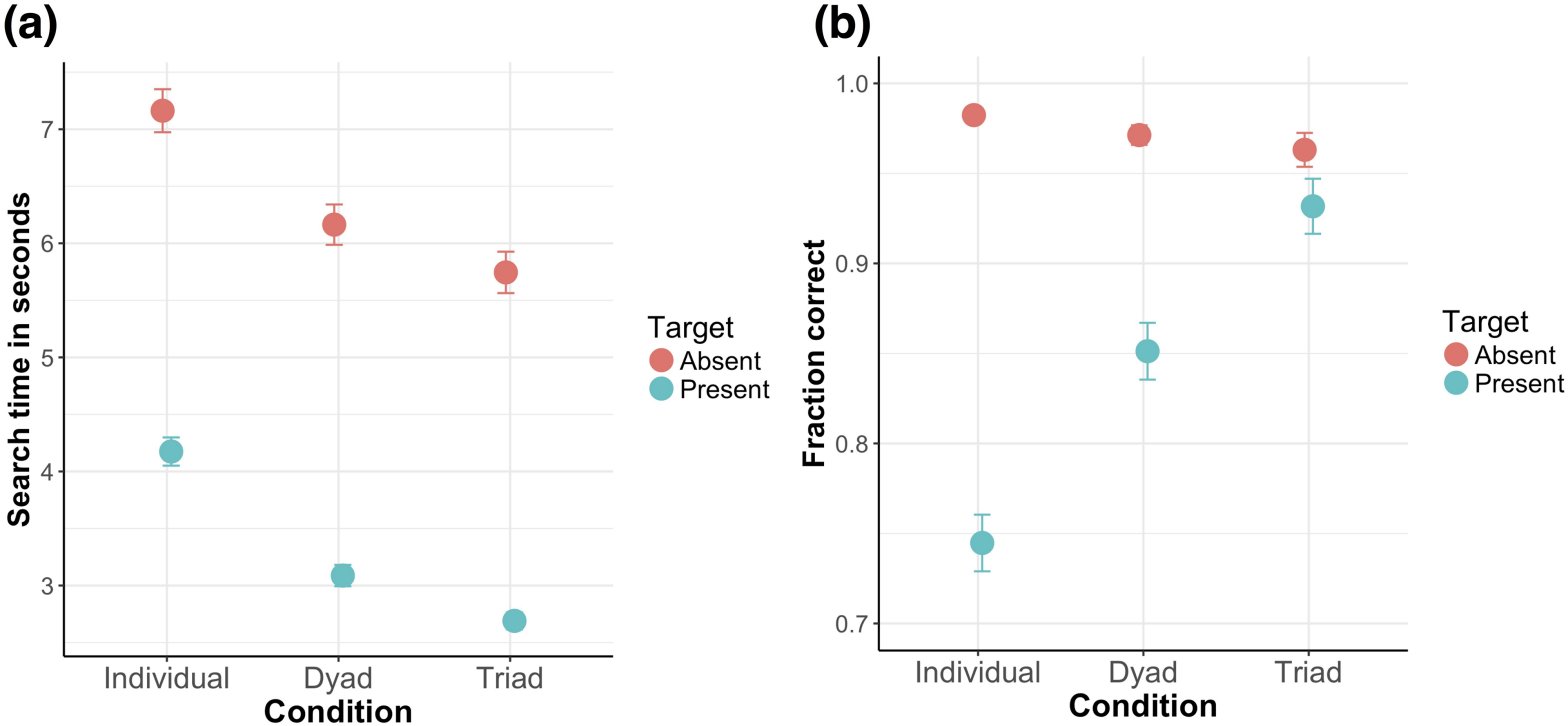
Descriptive overview. (a) Mean search time as a function of condition (i.e., searching individually, in a dyad, or in a triad) separately for target present and absent conditions. (b) Mean accuracy as a function of condition (i.e., searching individually, in a dyad, or in a triad) separately for target present and absent conditions. Error bars in all panels are standard error of the mean.

In Fig 2a & 2b, a descriptive overview of the mean performance (i.e., search time & accuracy) is shown as a function of the individual, dyad, and triad performance, separately for target present and absent conditions. Note, for this overview, we first computed the average of the individual and dyad performances within a triad and then averaged across triads. In this overview, on a descriptive level, we observe that dyads search faster than individuals and that triads search faster than dyads. Moreover, for the target present condition, search accuracy tends to increase with group size while the opposite appears to be the case for the target absent condition. Note, however, for the target absent condition the accuracy approaches ceiling performance for all group sizes. We tested whether these observations are statistically reliable by applying a two factorial repeated measures ANOVA with the factors Group (Individual, Dyad, Triad) and Target (Absent, Present) for the dependent variables search time and accuracy. For both ANOVAs, we tested the assumption of sphericity using a Mauchly’s test for sphericity. If the assumption of sphericity was violated, we applied a Greenhouse-Geisser correction. For the search times, we find a significant main effect of Group (*F*(1.39, 26.32) = 213.30, *p* < .001) and Target (*F*(1, 19) = 627.88, *p* < .001) but no significant interaction effect (*F*(2, 38) = 0.19, *p* = .825). We followed up the ANOVA by pairwise post-hoc comparisons. For this purpose, we averaged across the levels of the factor Target and compared search times between the levels of the factor Group to test whether dyads outperform individuals and whether triads outperform dyads. We find that dyads perform the search significantly faster than individuals (*t*(19) = 14.32, *p* < .001), and that triads perform the search significantly faster than dyads (*t*(19) = 8.70, *p* < .001). We repeated this analysis for the dependent variable accuracy. We found a significant main effect for the factor Group (*F*(2, 38) = 26.84, *p* < .001) as well as for the factor Target (*F*(1, 19) = 142.80, *p* < .001) and a significant interaction effect (*F*(2, 38) = 39.83, *p* < .001). As post-hoc tests to test for group differences, we performed pairwise comparisons between the levels of the factor Group, separately for the two factor levels of the factor Target. For the target present condition, we found that dyads perform a more accurate search than individuals (*t*(19) = 4.89, *p* < .001), and that triads perform a more accurate search than dyads (*t*(19) = 4.14, *p* < .001). For the target absent condition, we found no significant differences between groups (*ps* > .09). In sum, our findings suggest that dyads do perform a faster search than individuals while triads do perform a faster search than dyads. For the target present condition and search accuracy, we find the same pattern of results: Dyads perform a more accurate search than individuals and triads perform a more accurate search than dyads.

**Fig 3:**
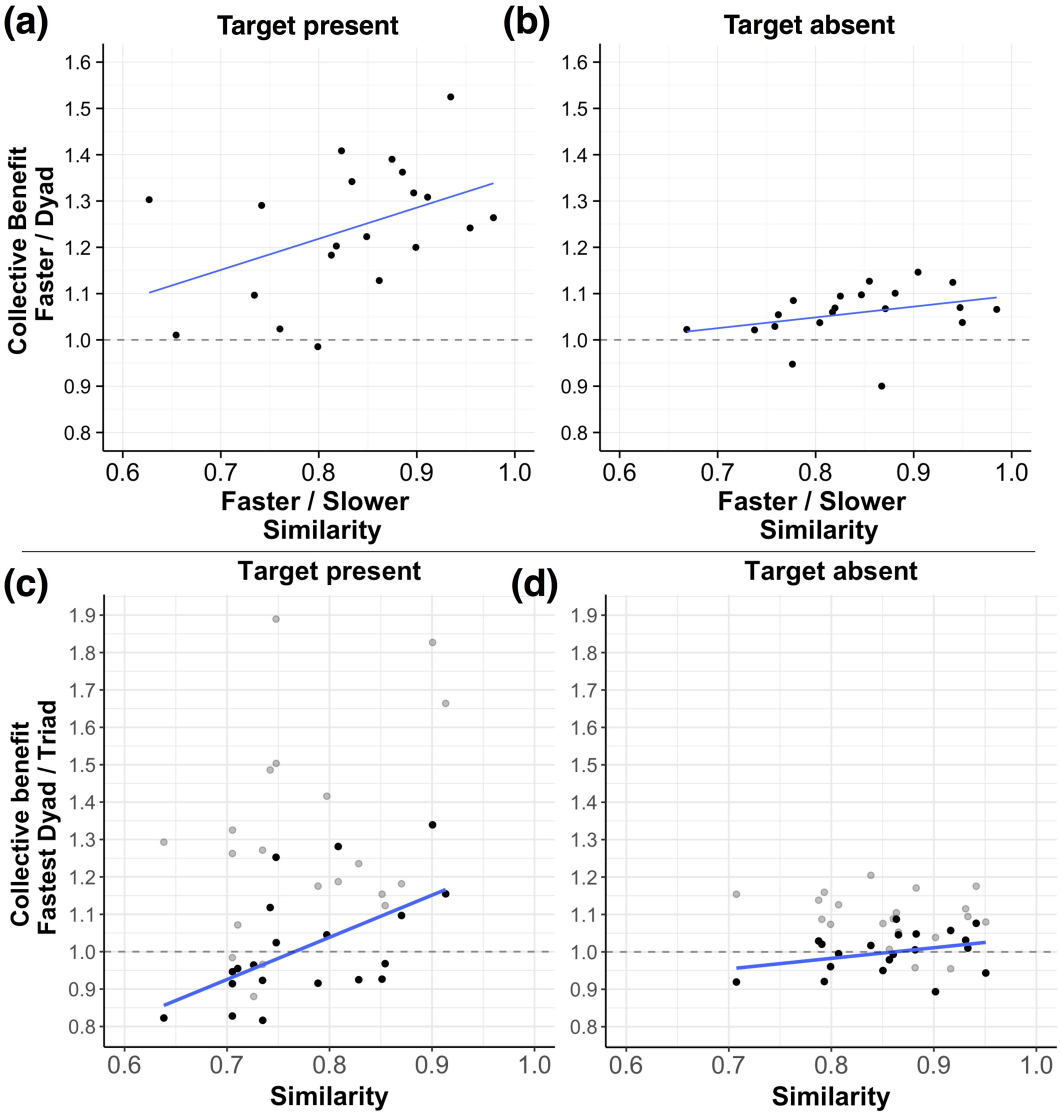
Collective benefit overview. The collective benefit is plotted as a function of similarity, separately for dyads ((a) Target present; (b) Target absent) and triads ((c) Target present; (d) Target absent). For the ordinate, a value above one indicates a collective benefit. For the abscissa, the closer the value is to one, the higher the similarity. Note, for panels (c) and (d), the light gray points indicate comparisons between the triad performance and the best individual performance in a triad.

To test whether dyads actually reached a collective benefit (i.e., outperformed the best member within a group) and whether interindividual similarities in performance are a predictor of the collective benefit, we calculated two measures. As a measure of the collective benefit, we divided the better individual’s search time by the dyad’s search time for correctly classified trials (i.e., participants correctly indicated whether a target was present or absent). For this measure, a value above one would indicate a collective benefit (i.e., that dyads outperform the better of the two individuals in a dyad). To test whether interindividual similarities in performance predict the collective benefit, we also computed a measure of the similarity of the individual search times for correctly classified trials. For this measure, similar to earlier studies [5, 14], we divided the best member’s performance by the worst member’s performance. For this measure, the closer the number is to one, the more similar the individual’s performances are. Note, however, that this measure does not take into account any search preferences of the individuals (e.g., whether co-actors tend to search in a similar search pattern). We calculated both these measures for each dyad, separately for target present and absent conditions and then averaged across dyads within a triad (for a descriptive overview, see Fig 3a & 3b). Note, we averaged across dyads within a triad to fulfill the statistical assumption of independent observations (i.e., performances of dyad pairings within a triad are dependent as each individual is involved in two dyad pairings). On a descriptive level, both for target present and absent conditions, dyads tend to outperform the better performance of the individuals. Moreover, the collective benefit tends to slightly increase as a function of the similarity score. We tested whether these observations are statistically reliable using t-tests and correlations. Using one sample t-tests and testing against one, we found that dyads reached a collective benefit both for target present (*M* = 1.24, *SD* = 0.14, *t*(19) = 7.61, *p* < .001) and target absent conditions (*M* = 1.06, *SD* = 0.06, *t*(19) = 4.45, *p* < .001). In addition, we found that the similarity of the individual performances does significantly predict the collective benefit for the target present condition (*r* = .44, *t*(18) = 2.11, *p* = .049) but not for the target absent condition (*r* = .33, *t*(18) = 1.46, *p* = .161).

To address the possibility that dyads achieved a collective benefit at the expense of performing a less accurate search (i.e., did not correctly indicate whether the target was present or not present), we tested whether dyads outperformed the better of the two individual performances also for the search accuracy, separately for target present and absent conditions. For the target present condition, we found that dyads performed a more accurate search on a descriptive level—however, their performance did not reach significance when performing a one sample t-test and testing against one (*M* = 1.03, *SD* = 0.11, *t*(19) = 1.33, *p* = .198). For the target absent condition, however, we found that dyads performed a significantly *less* accurate search (*M* = 0.98, *SD* = 0.03, *t*(19) = −4.11, *p* < .001).

In sum, these result suggest that the search time advantage for dyads for the target present trials was not achieved at the expense of performing a less accurate search than individuals. For the target absent condition, dyads searched faster only at the expense of performing a less accurate search. Yet, we want to note that dyads still perform a highly accurate search, which is close to ceiling performance. Moreover, similarities in the individual performance are a predictor of the collective benefit for the target present condition.

To test whether triads can outperform the best dyad pairing in a group (i.e., attain a collective benefit) and whether similarities in performance between dyads predict the collective benefit of triads, we again computed two measures to quantify the collective benefit and the similarities in performances between the dyads, respectively. To quantify the collective benefit, for each triad, we divided the search time of the best dyad in a triad by the search time of the triad. Again, a measure above one would indicate that triads outperform dyads. Second, to quantify the similarities between dyads within a triad, we computed ratios between each dyad pairing (i.e., divided the faster dyad’s performance by the slower dyad’s performance) and averaged across these ratios for each triad. Again, the closer this ratio is to one, the more similar the performances of the dyads were. We calculated these measures for each triad for correctly classified trials, separately for target present and absent conditions (for a descriptive overview, see Fig 3c & 3d). On a descriptive level, both for target present and absent conditions, only about a third of the triads outperform dyads and these triads tend to have dyads which perform more similarly. These observations suggest that triads generally did not reach a collective benefit but that similarities in the dyad’s performances still tend to predict the collective benefit. We tested whether these observations are statistically reliable. In line with these observations, using one sample t-tests and testing against one, we neither found a collective benefit for the target present (*M* = 1.01, *SD* = 0.15, *t*(19) = 0.32, *p* = .752) nor target absent (*M* = 1.00, *SD* = 0.05, *t*(19) = −0.07, *p* = .943) condition. Note, however, when comparing the triads’ performances to the best members’ in the group (see light gray points in Fig 3c & 3d), we found that triads still outperform the best individual member of a group, both for target present (*M* = 1.29, *SD* = 0.27, *t*(19) = 4.88, *p* < .001) and target absent (*M* = 1.09, *SD* = 0.07, *t*(19) = 6.12, *p* < .001) conditions. With regard to the measure of the performance similarities, we found that the similarities in performance between dyads still significantly predict the collective benefit for the target present condition (*r* = .55, *t*(18) = 2.83, *p* = .011). However, this is not the case for the target absent condition (*r* = .33, *t*(18) = 1.49, *p* = .154). We also tested whether triads performed a more accurate search than dyads. In line with the results on the dyads’ collective benefit above, using one sample t-tests and testing against one, we found that triads did not significantly perform a more accurate search when a target was present (*M* = 1.02, *SD* = 0.13, *t*(19) = 0.76, *p* = .456). As above, for the target absent condition, we found that triads actually performed a significantly less accurate search (*M* = 0.97, *SD* = 0.04, *t*(19) = −3.96, *p* < .001). Again, however, we want to note that the triads’ accuracy is close to ceiling performance. In sum, these results suggest that triads did not reach a collective benefit (i.e., did not outperform the best dyad pairing within the triad) but that similarities in performance are still a predictor of the triads’ performances. Moreover, triads still do outperform their best member in the group.

**Fig 4:**
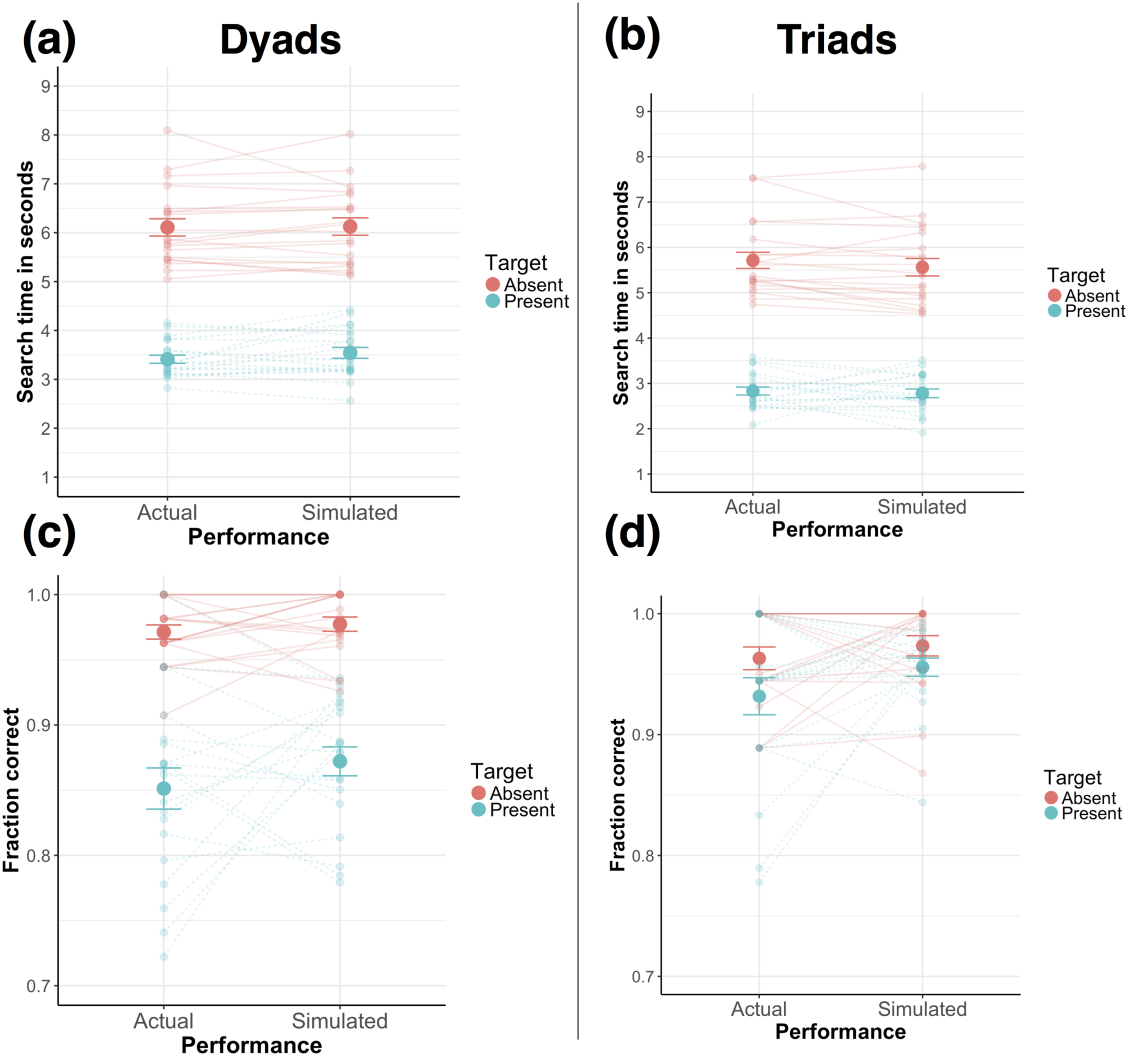
Comparison of simulated and actual performances. Simulated vs. actual search time (in seconds) for target present and absent conditions, separately for dyads (a) and triads (d). Predicted vs. actual search accuracy as a function of target present and absent conditions separately for dyads (b) and triads (c). Error bars in all panels are standard error of the mean. Lines in lighter colors show the non-aggregated data.

Given that members of a group do not have any means to communicate information within a trial to coordinate their joint search (i.e., devise a division of labour strategy [4, 19]), we also predicted that members of a group act independently of each other. Given this prediction, we tested whether the search performance by dyads and triads would match a simulated independent performance [4, 19]. For this purpose, we simulated a hypothetical dyad and triad performance under the assumption that the members of a dyad or triad acted independently (i.e., participants did not change how they performed the search task depending on whether they were alone, in a dyad, or in a triad). For this simulated independent performance, for dyads, we randomly sampled the performance (i.e., search time and accuracy) of a trial from each dyad member’s individual trials. We then compared these performances and selected the faster of the two search times and the corresponding accuracy of the faster search time (i.e., whether the trial was correctly or incorrectly classified as target absent or present). The rationale behind this sampling is that if members of a dyad would independently perform the search task, the faster of the two members would press a key first to indicate whether a target is present or absent, thereby ending the trial. We performed this random sampling for a 1000 iterations for each dyad, separately for target present and absent trials. We averaged across the sampled search times and accuracies for each dyad, separately for the target present and absent conditions. We repeated this procedure also for triads. In order to estimate the triads’ independent performances, we sampled search times and accuracies from all three individual performances and selected the fastest search time and the corresponding accuracy. We then assessed whether the simulated independent performance matches the actual dyads’ and triads’ performances. On a descriptive level, we found that the stimulated search times match the actual search times of dyads and triads closely (see Fig 4a & 4b), both for target absent and present trials. With regard to the search accuracies, the simulated averaged performance does match the actual performance both for dyads and triads (see Fig 4c & 4d). With regard to the dyads, we tested whether the actual performance significantly differs from the simulated performance using a 2x2 repeated measures ANOVA with the factors Target (Absent, Present) and Performance (Actual, Simulated). For the search times, we found no significant main effect of Performance (*F*(1, 19) = 1.30, *p* = .268) or interaction effect (*F*(1, 19) = 1.03, *p* = .322). We only found a significant main effect of Target (*F*(1, 19) = 447.82, *p* < .001). We found the same pattern of results also for the search accuracy. That is, we found no significant main effect of Performance (*F*(1, 19) = 1.74, *p* = .203) or interaction effect (*F*(1, 19) = 0.58, *p* = .457). We only found a significant main effect of Target (*F*(1, 19) = 120.00, *p* < .001). For the triads, we performed the same analyses, again using a 2x2 repeated measures ANOVAs with the factors Target (Absent, Present) and Performance (Actual, Simulated) and found the same pattern of results. That is, with regard to the search times, we found no significant main effect of Performance (*F*(1, 19) = 1.94, *p* = .179) and interaction effect (*F*(1, 19) = 0.42, *p* = .523), and a significant effect of Target (*F*(1, 19) = 360.14, *p* < .001). For the search accuracy, we found no significant main effect of Performance (*F*(1, 19) = 2.89, *p* = .106), Target (*F*(1, 19) = 3.65, *p* = .071), and no significant interaction effect (*F*(1, 19) = 0.56, *p* = .450). In sum, these results indicate that for dyads as well as for triads the simulated independent performances closely match the actual joint performances.

## Discussion

In the present study, we investigated how well groups of different sizes (individuals, dyads, or triads) perform in a joint visual search task, in which participants had no means to exchange any information. We tested whether dyads outperform individuals and whether triads outperform dyads. Examining the averaged performances, we found that dyads indeed perform a faster search than individuals and triads perform a faster search than dyads. While the performance benefit for dyads does replicate earlier findings by Brennan and colleagues [4], the performance benefit for triads is a novel finding, which suggests that increasing the group size does lead to additional benefits. Moreover, we tested whether dyads achieved a collective benefit. That is, we investigated whether dyads searched faster than the best group member’s individual search performance. We found that dyads did reach a collective benefit when a target was present and when it was absent. In the latter case, dyads reached this speed advantage at the expense of performing a slightly less accurate search than individuals. Search accuracies for the target absent condition, however, were close to a ceiling performance across group sizes. That is, dyads still performed a highly accurate search in the target absent condition. These findings extend earlier findings for joint visual search tasks [4] by showing that dyads can not only achieve a group benefit but also can achieve a collective benefit in a joint visuospatial task, in which they were not allowed to exchange any information. In particular, we have shown that dyads outperform the best member of the two members of a dyad. Due to the between-subject design in an earlier study [4], this aspect could not be addressed before. The fact that dyads performed a slightly less accurate search than individuals when a target was absent could be explained by the lack of information exchange. That is, members of a dyad had no means to agree on stopping the search (i.e., that a target was not present), possibly leading to searches that were stopped prematurely. Interestingly, in a recent study [15] in which dyads were allowed to verbally communicate, dyads did reach a collective benefit regardless of whether a target was present or not, highlighting the importance of an information exchange during a joint visual search.

In addition, we also investigated collective benefits for larger group sizes (i.e., triads). That is, we investigated whether triads reach a higher search performance than the best dyad pairing within a triad. In contrast to the results above, we found that triads did not reach a collective benefit, indicating that the collective benefit found for dyads does not necessarily scale up to larger group sizes. As noted above, based on the averaged performances, triads still outperform dyads and we also found that triads outperform the best member of the group. Yet, triads do not outperform the best dyad combination within the triad. Future studies are needed to verify whether this result cannot alternatively be explained by a ceiling effect. That is, a future study could investigate whether the collective benefit for triads relative to dyads might be present for larger stimuli set sizes compared to smaller ones. In addition, when a target was absent, triads actually performed a less accurate search than dyads. Again, it should be noted that triads still performed a highly accurate search close to a ceiling performance. Given that dyads already performed a less accurate search than individuals, these findings suggest that the severity of the lack of information exchange increases with group size.

Apart from investigating whether dyads and triads achieve a collective benefit, we also investigated whether the collective benefit can be predicted by the similarity of the individuals’ and dyads’ performances, respectively. With regard to the dyads’ collective benefit, we found that the similarity of the individual performances significantly predicted the dyads’ collective benefit when a target was present. Similarly, for the triads’ collective benefit and when a target was present, we found that the similarity of the dyads’ performances significantly predicted the collective benefit for triads as well. These results extend earlier findings that showed that similarities of the individual performances predict the collective benefit for a joint visuomotor task [14] and a joint perceptual decision-making task [5], suggesting that similarities between the individual performances could be a general predictor for collective benefits across several joint tasks. In addition, finding this effect also for triads suggests that similarities in performance could also be a predictor for collective benefits for larger groups performing joint visuospatial tasks. Future studies could aim to quantity similarities of the individual performances on a more detailed level. That is, a future study could relate similarities in how participants search individually to each other. That is, co-actors might have similar search patterns when performing the search task alone (e.g., both co-actors start searching on the left half of the screen and then move to the right half of the screen). Quantifying similarities on such a more detailed level might lead to different results as for instance more complementary search patterns could lead to a higher joint performance than similar search patterns. Moreover, future studies could investigate whether similarities in performance are also a predictor of the collective benefit in other joint cognitive tasks such as memory tasks [20, 21] or problem-solving tasks [29, 30].

When a target was absent, however, the similarities in performance did not significantly predict the collective benefit neither for dyads nor triads. Given the reduction in search accuracy for dyads and triads for target absent trials, respectively, it could be that the search times for this condition do not fully reflect how long participants would have taken to accurately complete the search. Therefore, we suspect that the correlations could be over- or underestimated in the current study. A future study could address this point by allowing groups to exchange information (e.g., verbally communicate) while performing the joint visual search task and test whether similarities in performance predict the collective benefit when a target is absent. Moreover, finding no significant correlation for target absent trials could be informative to understand under which circumstances similarities in performance are a predictor of the collective benefit and when this is not the case. In earlier studies that did find that similarities in performance predict collective benefits, co-actors were allowed to exchange information (i.e., either verbally [5] or they saw the effect of their co-actors’ actions on the computer screen [14]). Here, we only found that similarities in performance are a predictor for target present but not for target absent trials. Moreover, we argued above that the severity of the lack of information exchange was at least more pronounced in target absent trials as co-actor’s had no means to agree on when to stop the search. Taken together, we suggest that similarities in performance are a predictor of collective benefits in joint tasks in which either information is exchanged or in which there is no need to exchange information. Future studies could further investigate how the relation between individual performance similarities and collective benefits depend on an information exchange between co-actors in a joint task.

Finally, as dyads and triads were not allowed to exchange any information in the present study, we predicted that group members would act independently (i.e., they are not collaborating). To test this prediction, we simulated dyad and triad performances based on the individual performances, which assumed no interaction between members of a group [15, 19]. We found that the simulated performances closely match the dyads’ and triads’ actual performances, suggesting that members of a group indeed act independently for the present search task. Given that in the present search task members of a group did not have any means to exchange information during a trial to coordinate their joint search, this result validates our simulated independent performance. Moreover, the present study provides a basis for future studies which allow an exchange of information between co-actors. That is, future research could further investigate how an exchange of task-relevant information during a trial increases joint performance relative to a simulated independent performance [15, 19]. With such a comparison, it can be differentiated how much of a collective benefit can be attributed to an actual collaboration between members of a group and how much of the benefit can be attributed to the mere performance benefit due to jointly performing a task. Moreover, we want to emphasize that in the case of dyads, participants still reached a collective benefit even though members of a group did act independently. In line with earlier findings [19], findings of the present study suggest that groups can already reach a collective benefit in a joint visuospatial task, in which members of a group act independently (i.e., do not collaborate).

As a point of note for future studies, the present study did not investigate how the social presence of co-actors affects joint performance. In the study by Brennan and colleagues [4], participants sat in separate rooms while in the present study participants sat next to each other. Work on social facilitation effects has investigated whether the mere presence of another person which does not perform any task can affect performance (e.g., [31]). A future study could address whether the simulated independent performance also matches the actual joint performance in a setting in which co-actors sit in separate rooms.

In sum, the present study extends earlier findings on joint visual search by showing that dyads can achieve a collective benefit in the absence of any information exchange. However, these findings do not necessarily generalize to larger group sizes (i.e., triads) as no collective benefits were found for triads. That is, triads did not outperform the best dyad combination within the triad. Yet, triads still reached a higher performance than dyads and the best individual member in a triad, suggesting that increasing the group size still leads to group benefits. Moreover, the similarities in performance are a predictor of the collective benefit regardless of whether dyads or triads perform a joint visual search task. The latter finding combined with earlier studies [5, 14] suggests that similarities in performance could be a general predictor of collective benefits across joint tasks. Moreover, findings suggest that the joint performance of dyads and triads in visuospatial tasks in which no information is exchanged can be well simulated based on the individual performances. The present study therefore provides a basis on which future studies, in which information between co-actors *is* exchanged, can built on. That is, future studies can test to what extent allowing an exchange of information can improve joint performance beyond a simulated independent performance.

## Materials and methods

### Participants

60 students (39 female, *M* = 22.5 years, *SD* = 3.4 years) of the University of Osnabrück participated in this study, which were recruited in 20 sets of triads. All participants had either normal or corrected to normal vision. The study was approved by the ethics committee of the University of Osnabrück. We informed participants about their rights and all participants signed a written consent form. Participants either received a monetary reward or course credits for participation.

### Experimental setup

The experimental setup consisted of three computer screens (DELL U2713Hb, 2560 x 1440, 60Hz) placed next to each other, which were separated by wooden dividers. Participants sat at a distance of 100 cm in front of the computer screens and a response box was placed in front of them (The Black Box ToolKit). In order to minimize external noise, each participant wore noise-canceling headphones. The experiment was programmed using Python (2.7.3).

### Experimental procedure

In the experiment, participants performed a visual search task individually, in dyads or in triads in a within-subject design. Prior to the experiment, each co-actor was assigned an avatar (blue square, purple triangle, or red circle) and preceding each trial the number of co-actors was indicated (i.e., whether participants were required search alone, in dyads, or in triads). That is, members of a triad were always aware whether they searched alone, in a dyad or in a triad. In a trial, participants were required to search for a circular target object among 36 circular objects (radius: 36 pixels / 0.48 visual degrees) within a rectangle subset of the screen (1680 x 1260 pixels, 22.51 x 16.88 visual degrees). The objects were randomly distributed across the screen (minimum distance between objects 150 pixels / 2.01 visual degrees). The target and distractor objects differed in one feature: To each distractor a small antenna (length: 4 pixels / 0.05 visual degrees) was attached, either orientated in the 0, 90, 180, or 270 degrees direction, while no antenna was attached to the target object. Participants’ task was to indicate whether the target was present or absent using the response boxes. They pushed a green button if a target was present or a red button if the target was absent. Once participants responded, they received performance feedback (i.e., whether their response was correct or not) and which co-actor responded. In half of the trials, the target was present. We instructed the participants to perform the search quickly and accurately. Note, when participants performed the search in dyads or triads, the first response within a group terminated the search and only counted to performance. Each participant performed 144 trials—36 for each combination of co-actors and 36 individual trials. Conditions (i.e., whether the task was performed alone, in dyads, or in triads) were pseudorandomly interleaved (i.e., repetitions of conditions in consecutive trials were avoided). The experiment took about 40 minutes. Note, participants were not allowed to communicate throughout the experiment and could not see the other participants’ computer screens (i.e., wooden occluders were placed between computer screens).

## Supporting information

S1 File Zip file containing datasets underlying Figs 2, 3, and 4. (ZIP)
